# Conversation Analysis and genre theory

**DOI:** 10.3389/fsoc.2023.1258672

**Published:** 2023-12-11

**Authors:** Ruth Ayaß

**Affiliations:** Faculty of Sociology, Bielefeld University, Bielefeld, Germany

**Keywords:** genre theory, sociology, Conversation Analysis, social theory, communicative genres

## Abstract

Since its genesis in the 1960s, Conversation Analysis (CA) has noticeably developed further in terms of its subjects and methods. Its analyses, today, not only focus on conversations in the original sense, but also on visual elements such as gazes in interactions and the role of bodies. However, it also analyzes especially larger communicative units, e.g., in institutionalized settings and it addresses larger sequences of action. One of these approaches is the theory and analysis of *communicative genres*. Communicative genres are to be understood as consolidated forms of communication. The theory of communicative genres understands these forms as solutions to communicative problems. Genre analysis is methodologically grounded in CA; however, it exceeds it conceptually and theoretically, thus anchoring its questions clearly within sociology. The paper starts out by outlining the concepts and theory of communicative genres. The article discusses the empirical contribution of genre analysis using the example of three so-called “families of genres” families. The examples discussed are reconstructive genres (speaking about the past), genres of moral communication (speaking about other people’s behavior), and projective genres (speaking about the future). Using examples from empirical research, it is shown which communicative problems these genres solve. The paper finally considers the insights to be gained from genre analysis for sociology and CA.

## 1 Introduction

Since Conversation Analysis (CA) turned to recording *natural* conversations in the 1960s and thus focused on the real procedure of everyday interactions, there has been a noticeable sophistication of the approach.^1^ This has taken place in several areas. (1) Firstly, CA, as a method, has become largely independent from sociology, from which it originally emerged, and has established itself as a method in other disciplines. CA has been used with major results in psychology, anthropology and linguistics, where it has resulted in a shift in understanding spoken language. (2) Secondly, CA, in many ways, is no longer an ‘analysis of conversations’ (if it ever was). Thanks primarily to the possibility of gathering visual data provided by the availability of video recording, it turned to analytical elements beyond ‘conversation’ in the original sense, i.e., the gaze behavior of interactants, the handling of artifacts in interaction, the orientation of bodies in relation to each other and their positioning in space, and a number of other features. (3) Finally, CA, which originally focused on a turn as a construction unit and the transition of these turns in interactive exchanges, has taken larger units of analysis into view. This applies, for instance, to settings in which complex units of action can be found, e.g., courts of justice (cf. for example Atkinson and Drew, 1979), but also to the connection of CA proper with cultural practices and thus with ethnographic questions (cf. especially Moerman, 1988).

The analysis of communicative genres is a part of these approaches which address larger units of communication. It emerged in the German-language sociology of the 1980s and essentially originated with Thomas Luckmann and Jörg Bergmann. While CA seems to be mostly interested in micro-scale forms of interaction – recall essay on “Some uses of ‘uh huh’ and other things that come between sentences” (Schegloff, 1982) or the analysis “What’s in a ‘nyem’?” (Jefferson, 1978) – the analysis of communicative genres, from the beginning, has been concerned with larger-scale communicative forms and addresses complex communicative problems, e.g., speaking about the past, moralizing about other people and their (good or bad) behavior, or planning one’s future.

The theory and analysis of communicative genres is an approach which aims to provide a theoretically well-founded empirical analysis of consolidated structures in everyday communication (Luckmann, 1986, 1989; Bergmann and Luckmann, 1995). The term ‘communicative genre’ refers to a theoretical concept rooted in the sociology of knowledge. Communicative genres are thus *consolidated forms of communication on which interactants can rely for reciprocal orientation*. These forms are stored in the subjective stock of knowledge and can thus be retrieved from this stock, can be updated and are familiar to the speakers.

Genre analysis is the *empirical* analysis of these communicative forms. From the beginning, the concept has been understood as a guiding question for empirical research. Genre analysis is concerned with questions such as: How do these forms take place in terms of their sequential structure? How do they begin and how do they end? Who participates in them and in which context do they occur? What are the constitutive elements for the implementation of a genre? What are the optional ones? Which outer and inner forms do genres take? Crucially, genre analysis is not restricted to linguistic analyses of these communicative forms. Instead, genre analysis seeks to understand the social function of the forms as well as the question of their sociological classification. Genres are characterized by a relative rigidity, which differentiates them from the rather ‘spontaneous’ communicative acts. Spontaneous forms and genres together make up the communicative budget of a society. To describe this “communicative budget” (Luckmann, 1986, 206; Bergmann and Luckmann, 1995, 300) is the aim of genre analysis.

Genre analysis takes CA as a methodological starting point, however exceeds it in several respects. The present paper seeks to demonstrate which insights both sociology and CA can draw from genre analysis. It will first outline the theory of communicative genres, explaining the theoretical background and central concepts (section 2). Section 3 is then dedicated to a detailed description of the empirical objects of the approach. The analysis is based on the examples of three “families of genres,” i.e., reconstructive genres, genres of moral communication, and projective genres. For these families of genres, empirical examples of individual genres are provided, e.g., gossip as an example of reconstructive genres. The fourth and final section will discuss the status of genre analysis with respect to sociology. It shows how genre analysis can open a pathway to sociological theories and especially social theories.

## 2 The theory of communicative genres

### 2.1 Emergence of the approach

For the development of the approach, dialogues with linguistics, anthropology, literary studies, ethnology and ethnography (of speaking) played an important role. From these disciplines, the theory of communicative genres received essential impulses; at the same time, it delimits itself from them and exceeds them. The concept of genre emerges, for instance, in narrative analysis in linguistics (e.g., Labov and Waletzky, 1967) and its studies of the structure of oral narratives. Based on this, the analysis of communicative genres was concerned with the situative embedment of the communicative forms as well as the interactive generation of genres. Another discipline which genre research draws on is linguistic anthropology, notably ethnography of speaking. Ethnography of speaking was explicitly concerned with empirical forms of oral communication in non-western cultures. Hymes coined the term *speech event* for this, a term not unlike that of genre. Oral ethnography has produced a large number of studies describing speech events of this type [the empirical studies in Gumperz and Hymes (1972) and Bauman and Sherzer (1974)]. Hymes, too, in his distinction between “means of speaking” and “speech economy” sought to establish a theoretical framework for analyzing the communicative repertoire of various individual local societies (Hymes, 1974).

Another impulse comes from the work of the Russian literary scholar Mikhail Bakhtin. The significance of his work on “speech genres” stems from the fact that – aside from discussing works of literary fiction such as Dostoevsky and Rabelais – he engaged with genres of oral communication already in the 1950s.

“Speech genres organize our speech in almost the same way as grammatical (syntactical) forms do. We learn to cast our speech in generic forms and, when hearing others’ speech, we guess its genre from the very first words; we predict a certain length […] and a certain compositional structure; we foresee the end; that is, from the very beginning we have a sense of the speech whole […].” (Bakhtin, 1986, 78/79)

Unlike Bakhtin (1986, 78) however, genre analysis in sociology does *not* make the assumption that all speaking takes place in communicative genres. Classical philology and comparative literary studies have also played a role in the genesis of genre as a concept: These disciplines studied the oral tradition of the past already in the 1920s, e.g., in Milman Parry’s analyses of the formulaic structures of Homerian metrics (Parry, 1971) or Albert B. Lord’s analysis of the epic structures of the songs in the *Iliad* and the *Odyssey* (Lord, 1960).

The analysis of communicative genres is rooted in all these approaches, and yet it develops the concept of genre further. The original understanding of communicative genres is that they are fixed forms of oral communication. It is the aim of this approach to describe genres empirically – in terms of their internal features, their constitutive and variable elements, their sequential procedure, their (external) features, e.g., structure of participants, their situative integration in the interactive context.

### 2.2 Communicative genres as solutions to communicative problems

What do communicative genres accomplish in everyday life? What function do they serve? Wherever humans carry out certain activities regularly – be it voluntary or out of necessity – they do not come up with new means to achieve their goals each time; instead, they rely on fixed forms or patterns which have proved useful. Such patterns have several advantages: On the one hand, they provide a solution for whatever needs to be accomplished that has proved to be successful at least once; in other words, they offer a realistic possibility that what is to be accomplished is actually feasible. On the other hand, such recurring patterns give stability to social situations: One does not have to go through the trouble of thinking things through from the beginning and decide what to do; one can simply rely on the established pattern. Patterns relieve actors of the decision-making burden. However, it can also be said of the other participants in the interaction that they can let themselves be guided by what is well-tested and thus know what to expect. Reliance on patterns provides actors with behavioral security. Such fixed forms are found in all societies at all times. They structure social life, and they are the basis for processes of institutionalization (Berger and Luckmann, 1966, 47–92 for details). This can be illustrated with a simple and ‘small’ example from ritual communication: Interactants know what a greeting is, they also know when it is carried out, when it is expected, and who should be greeted. They also know that a greeting requires a certain reaction, even though they may not be familiar with the concept of reciprocity. And they know that failure to greet or ignoring a greeting is seen as bad manners and can be interpreted as arrogant or impolite demeanor. Greetings are a striking example in the context of and in comparison with communicative genres because of their high degree of consolidation as rituals of interaction, which exceeds that of genres (see Goffman, 1967). Patterns and fixed forms come in different degrees of bindingness, and they can pertain to different areas of social life. They are mostly described as routines, rules or rituals. Genres, in comparison, leave interactants more options for modification. It is against this background that the function of communicative genres becomes clear: Communicative genres are consolidated forms of communicative action on which interactants can rely on in managing recurrent social situations. Thus, communicative genres have a double impact on interaction: On the one hand, they create an obligation; on the other hand, they reduce the burden on interactants (Luckmann, 1986, 204). Another aspect which shows the sociological impetus is Luckmann’s comparison between genres and institutions:

“In certain aspects communicative genres resemble social institutions. But social institutions are routinized, more or less obligatory solutions to *elementary* problems of social life. They regulate functionally very clearly definable kinds of social interactions such as production, reproduction, the organization of power, etc. Communicative genres, on the other hand, offer solutions to specifically *communicative* problems.” (Luckmann, 1992, 227)

Genres, that is, are consolidated solutions to recurrent communicative problems. Luckmann’s writings repeatedly show that the concept of the communicative genre raises questions pertaining to the *sociology of knowledge*. It acts upon the “general structure of communicative processes (…) in which stocks of knowledge of varying levels of explicitness are transferred” (Luckmann, 1986, 194, our translation). The analogy with institutions as well as the observation that communicative genres are anchored in the stocks of knowledge of the actors shows that genre analysis seeks to achieve more than a description and analysis of the linguistic and communicative elements of genres. The goal is to utilize the analysis of genres as a gateway to statements about society. The comparison with institutions, here, is seen as an *analogy*. With this analogy in mind, communicative genres can be understood as “‘institutions’ of *communicating* about life, including social life, within social life” (Bergmann and Luckmann, 1995, 290).

However, just as the concept of the communicative genre has a fixed sequential structure and a specific constellation of participants, it also inherently provides a solution to a *particular problem*. Greetings, for instance, on the one hand generate and show mutual perception, and on the other hand help re-establish and affirm the social relation between the interactants for each other. We greet our manager in a different way than we greet the mailman at our door, but we also greet someone in our family differently depending on whether we see them at the regular family supper or whether a year has passed since the last encounter or one of us has been on a dangerous journey. Who greets whom and when is part of the knowledge of everyday practices which interactants possess. It is stored in the stock of knowledge of interactants in everyday life.

“In every society there is the elementary problem of the way in which events, issues, knowledge, and experiences can be thematized, arranged, managed, and handed down in an intersubjectively binding way and under different criteria of meaning. For these problems – just as for the elementary problems of securing subsistence, preservation of the species, socialization, conflict regulation or the formation of structures of domination (*Herrschaftsbildung*), there must be organized, that is nonaccidental, solutions.” (Bergmann, 1993, 29)

Genres provide such established communicative solutions to communicative problems on the level of everyday interaction.

### 2.3 Concepts and method in genre analysis

The *method* used here is CA, which also has its origin in sociology, more specifically in ethnomethodology. This methodological orientation places the focus on the sequential structure of communicative genres and on their concrete situational forms of realization. For genres are constituted interactively, and CA provides the means to analyze the intersubjective dimension of interaction.

For the empirical procedure of genre analysis, two levels of analysis are relevant: The *internal* and the *external* structure (Luckmann, 1986, 203ff.; Bergmann and Luckmann, 1995, 291ff.). The internal structure consists of the communicative elements used by the interactants for the concrete realization of the genre: Rhetorical and stylistic devices, rhythms, phonetic melodies and other prosodic elements, semantics, lists, registers, etc. The internal structure, in some sense, provides the material for communicative genres. Genres also differ in the elements of internal structure that can be used (or at times must be used), how binding they are, and the position they take in the procedure of the genre. The internal structure is constituted by a range of elements whose level of obligation is determined by the genre. For instance, it is part of the reconstructive genre of gossip that the one gossiped about is mocked in the reconstruction of dialogs (see section 3.1); for genres of moral interaction, it is mostly elements of indirectness that have proven relevant (section 3.2); for projective genres the role of modal verbs (can, must, should) becomes salient (section 3.3).

Just as there is an internal structure, there is an external structure. The external structure comprises all the elements which determine the genre from the outside: The social situation, the social roles, the constellation of participants, and the communicative *milieu*. The external structure is the level through which features of the social structure of a society impact communicative genres. In this way, the external structure mirrors the socio-structural framework society sets for genres. The external structure is the level at which and through which society influences communicative genres: Through the social relations of the actors, through gender, social roles, age, status, etc. As a level of analysis, the external structure is relevant for the *sociological* basis of genre analysis: If genre analysis limits itself to the analysis of internal structure, it hardly goes beyond a linguistic analysis. So, both levels together determine the structure of a communicative genre. Günthner and Knoblauch (1995) have added a third layer aside from internal and external structure, thereby also highlighting the relevance of CA: The situative level. This *intermediary* layer consists of the concrete sequential, and situative patterns of a genre (the organization of turns, participation framework, etc.), i.e., all the interactive formats that are subject to studies in CA. In genre analysis, CA is the method *par excellence* for a sequential analysis of genres and thus an identification of the communicative problem solved by the genre.

Genres can be relatively independent and stand for themselves. Frequently (but by no means always), they are part of a *social occasion* which they belong to and which in turn frames additional genres. Genres, in such cases, can also be found in a foreseeable order. Answering the question of the typical contexts in which genres can be found ultimately involves describing the social occasion to which they possibly belong. Social occasions are understood as communicative units with relatively clearly defined spatial and temporal boundaries and typical participants’ roles. They contain more or less firmly structured action sequences which themselves at times can have various degrees of consolidation or institutionalization. An example of a social occasion is a conversation over a meal, a sales conversation, a party, a barbecue, a baptism or a funeral. In social occasions, specific genres now have a fixed place (a greeting, small talk, a prayer, a joke, etc.). It is not necessarily the case, however, that such social occasions are a fixed sequence of genres (even though some social occasions do have such a sequence; a prayer, for instance takes place at the beginning of a meal, but usually at the end of a service). Certain genres are part of certain social occasions, but at the same time, they are communicatively produced and framed *in* and *through* them. The term “social occasion” is also used by Goffman (1981, 165ff), in quite a similar sense (although of course Goffman does not provide a genre analysis in Luckmann’s sense). In describing the structure of lectures, Goffman repeatedly emphasizes how lectures are nested within the social situation and how the social situation, in turn, impacts the communicative form.

As Bergmann emphasizes, it was the “declared goal” of genre analysis from the very beginning “to elaborate a draft for a typology of communicative genres” (Bergmann, 2018, 290; our translation). This means that the analysis of communicative genres is also aimed at studying not individual genres, but various genres in relation to each other. This relation can take two different forms (on the following ideas, cf. Bergmann and Luckmann, 1995 and Bergmann, 2018).

(1) On the one hand, genres empirically present themselves in specific sequences and thus in their social contexts group into *clusters of genres*. In table talk, for example, different genres are produced in sequence and together constitute the *procedure* of table talk as a social occasion. The analysis of such clusters of genres is concerned with the ‘positioning’ of genres in their context: Which genres typically follow each other? Which do not and exclude a neighboring relation? For instance, making plans for something (say, a vacation) can transition into making plans for something else (say, the son’s sports activities) and finally into making meal plans together (see section 3.3). Such clusters can also be found in moral communication; for instance, when a gossiping conversation ‘is done’ with one victim, and it is now someone else’s ‘turn’ (see section 3.2). When interactants have successfully carried out a communicative activity, they carry on with it for some while. One is, so to speak, in planning mode (or in banter, gossiping, or joking mode) and maintains this until it is thematically or situatively exhausted. However, it is also possible for *different* genres to occur in a cluster. For instance, certain social occasions (e.g., a telephone conversation, a meeting, a lunch) often close not only with goodbyes but also with arrangements for the next meeting. Genres occur in a sequential organization, and their procedure is predictable for interactants.

(2) On the other hand, communicative genres can also be described systematically in terms of the *work* they complete and in terms of the problems they solve. Such resembling forms can be called *families of genres*. Several research projects concerned with different families of genres managed to demonstrate how different genres differ from one another, what they have in common, and what practical actions can be accomplished through them. (Section 3 discusses some of these families of genres in greater detail).

The entirety of these communicative forms which have consolidated into genres, together with the rather free forms of spontaneous communication, makes the “communicative budget” of a society (Luckmann, 1986).

“It should be obvious that under some circumstances almost any communicative process may have a bearing upon the maintenance – and transformation – of a society, but it is also clear that, in fact, some communicative processes are more important than others.” (Bergmann and Luckmann, 1995, 301)

Which processes those are cannot be determined *a priori*, but must be demonstrated by empirical work. Communicative budgets differ from society to society. This specificity of communicative budgets with regard to cultures or periods can be demonstrated through historical analyses and cultural comparisons. Valuable contributions in this respect are primarily the studies in ethnography of communication, which show what specific speech events look like in other cultures. In comparisons with the communicative budgets of other societies or the communicative budgets of other historical periods of our own society, it would thus be possible to show how these differ from each other, i.e., how they are communicatively ‘composed’ with regard to their genres. The analysis of the communicative budget is the ultimate goal of such endeavors.

## 3 On the analysis of communicative genres

It thus becomes apparent that communicative genres *by definition* have a common feature: They provide members of a society with patterns for solving communicative problems. Communicative genres, then, also differ from each other in what specific problem they act on (by representing a solution to it). In order to better describe the communicative function of specific genres and to determine which specific problem they solve, it is helpful to concentrate not exclusively on individual genres, but to study genres in the context of their families. What families of genres exist, how many members they have, and how they relate to each other are questions that can only be answered empirically. Focusing on *families of genres* in empirical research is a promising approach because, if nothing else, it provides a possibility for parallel analysis of communicative forms completing similar tasks. Raising such types of questions reveals the spectrum of communicative genres on which interactants can rely on in accomplishing their communicative tasks (for instance, an aggressive reproach instead of a teasing joke). It is plausible, in this respect, to assume that certain families of genres in one way or another occur in all societies – because they accomplish central tasks which are equally relevant in all societies. For Luckmann, reconstruction, moralizing and planning, among others, are examples of this type of families of genres (Luckmann, 2012, 35).^2^

On these three families of genres, there are empirical studies from various research projects which in the following sections will be introduced in more detail and placed in relation to each other. Each section is concerned with a specific family of genre – reconstruction (3.1), moral communication (3.2) and projection (3.3). The discussion is based on the theoretical and empirical results of three different projects funded by Deutsche Forschungsgemeinschaft DFG (German Science Foundation) which have analyzed communicative genres with a different main focus each.^3^

### 3.1 The communicative representation of the past: reconstructive genres

Remembering the past takes place on a number of levels in society, and it is often clearly visible in myths, tales, epics and histories of creation. However, reconstructions also take place in everyday life when people remember their own past and thus make it present communicatively through reconstructive genres. Reconstructive genres are the place where *past* experiences and events are worked upon. How societies represent the past, how they reprocess it, how they pass it on communicatively are essential questions for sociology. It refers not only to forms of remembrance practiced by countries, religions or organizations, but also to the practices of representing the past in everyday communication. In everyday communication, this happens for example in such diverse communicative forms as examples, media constructions, conversion narratives, or gossip. These (and other) reconstructive genres create reconstructions of past events and actions, and by extension almost always also of past *communicative* processes.

Using *gossip* as a case in point, form and function of reconstructive genres can be illustrated (for the following thoughts cf. Bergmann, 1993). In gossip, as is the case in other communicative genres, there is a transfer of *knowledge*. In the case of gossip, such knowledge consists of news about private affairs of someone who is known to the interactants. This knowledge must have novelty, and it is most suitable when it is in some way delicate, juicy, or indecent. This also means that one cannot get straight to the point. Bergmann provides an elaborate demonstration of how the subject of the gossip is established (carefully, because the recipient’s readiness to engage in gossip about the specific person must first be probed), and how the gossiping sequence then unfolds and is finally closed. It is especially this genre that is often found in sequences of genres: A story about the upstairs neighbor flows into another story about the same neighbor until the repertoire of news about this person is exhausted and one can turn to a new person (e.g., the downstairs neighbor). The communicative genre of gossip is characterized by a specific repertoire in its internal structure. Among those are hyperboles, which make the story entertaining and thus mark that which is told as worthy of being told by highlighting the ways in which the event was remarkable. Another constitutive element is the reproduction of speech, which is found in almost all reconstructive genres. Quoting or acting out entire dialogs add to the entertainment value and are often acknowledged with laughter. Most importantly, however, they allow the producer of gossip to mock the subject of gossip and use drastic wordings which are put in their mouth (pushing the responsibility for the choice of words on them).

However, which problem does this genre solve? The example of the reconstructive genre of gossip shows that the analysis of communicative genres is not restricted to the analysis of sequences or to the description of linguistic means. These are analytical steps *also* carried out by the analysis of communicative genres; however, it always poses the question as to which *problem* the specific genre solves. A constituent of gossip is the way in which its actors are placed in social relation. In gossip, one can immediately notice the participation structure taking the form of a “triad of gossip” (Bergmann, 1993): The social situation requires at least two interactants – one cannot gossip alone. And yet, there is inevitably a third person who plays a role: The subject of gossip, who is absent, but part of the triad. One can also not gossip about someone who is *present*. It is this absent subject of gossip – known to both interactants – about whom the producer of gossip now shares details they have somehow come across (but the recipient has not). Aside from this characteristic participation structure (i.e., the external structure) Bergmann also describes the typical features of gossip conversations: The subject of gossip generally must be introduced carefully and established as a topic of mutual interest. This mostly happens by bringing up innocuous details (“The- the Theissens moved out, huh?”; see Bergmann, 1993, 85 for this example). The gossiping actors rely on a typical inventory (i.e., a typical internal structure) which is required in reconstruction. Part of this inventory, for instance, is the reproduction of entire dialogs in which the questionable behavior of the subject of gossip is portrayed and judged. It is *also* part of this inventory, however, that participants portray themselves as ‘innocent’ witnesses who learned the details they are sharing without any action on their part. (“And Sunday morning I’m sitting on the toilet. Suddenly, I hear her again upstairs…”; see Bergmann, 1993, 126 for this example). In gossip, the participants solve the communicative problem of indiscretion. After all, there is a risk to talking badly about an absent person who is actually part of the social circle and whom one might meet again the next day.

Gossip is just *one* member of the family of genres of reconstruction. Many more communicative genres of everyday communication are part of it. Some are quite similar to gossip, some clearly differ from it. An example of another form of reconstruction is looking at photos together and reminiscing about the past (Keppler, 1994 on looking at family photographs together). Through the photographs, the shared family history is remembered, and events, names, and details are told and re-told, thus socializing new members into the family. In these reconstructive genres, the past is represented through communication and reprocessed for the present. And at the same time, gossip is a good example of how some communicative genres (but not all) can be members of more than one genre family. Gossip is not only a genre of reconstruction, but also a genre of moral communication.

### 3.2 Communicating respect and disrespect: genres of moral communication

Another family of genres are genres of *moral* communication. Moral communication is understood as forms of communication in which interactants speak about missteps and negotiate right and wrong behavior. The project studied the communicative means with which interactants communicate approval and respect (or disapproval and disrespect). The forms and genres used to express respect or disrespect vary greatly. They include compliments, reproaches, proverbs, complaints and stories of complaints, outrages, communication of stereotypes and lamentations (and, again, gossip) (see the contributions in Bergmann and Luckmann, 1999). What could be shown was that forms of moral communication are neither rare nor restricted to specific social occasions.

“Obviously, morality is omnipresent in everyday life; it is so deeply intertwined with everyday discourse that the interlocutors hardly ever recognize their doings as moral business.” (Bergmann, 1998, 281)

The ubiquity of moral communication shows that questions of right and wrong behavior can be articulated and communicated *situatively* by interactants, and that it is *through* and via this continual moral communication that interactants negotiate what should be seen as right and wrong behavior. What was found was that there is a preference for *negative* moral communication in our society, i.e., forms of disrespect and disapproval. Empirically, they can be found as reproaches and outrages, in mocking and lamenting and many other forms. A much rarer sight are forms of *positive* moral communication (compliments, excitement, etc.). Forms of speaking positively about others are far less frequent as well as far less sophisticated. In comparison with the many forms of negative moral communication, the few positive forms seemed either outdated (e.g., proverbs) or formalized (e.g., laudations) or, as was seen for instance in compliments, they were objectified beyond recognition (“Nice shoes!”). Modern societies are characterized by a decline of traditional values and living conditions and by processes of privatization, pluralization and individualization. These processes have also caused a “pluralization of morals” (cf. Bergmann, 1998, 290–292 for details). Thus, interactants cannot depend on *one* universal moral code, and instead must produce it interactively. What is right and wrong evidently cannot be taken as given. It is thus easier for interactants to communicate situatively and selectively about what is *not* acceptable than about what is acceptable. In sum, this means that interactants engage more with what they disapprove of – what they judge, see as wrong, and reject. Thus, the data material was fraught with reproaches, indignations, rants and other forms of moral communication.

There is, to be sure, a difference depending on whether the person being moralized is present or not. Moral communication can target absent people: For gossiping, ranting, etc. it is necessary that the person being talked about is absent. Moral communication, however, can certainly also address *present* people, e.g., by teasing, being indignant or in making accusations. The distinction between the *moral addressee* and the *communicative addressee* has proven crucial in the analysis of moral communication. This is especially the case when the communication is about someone who is *absent*. For instance, when re-enacting someone else’s indignation, when reporting about reproaches or when complaining about someone, interactants bring up someone’s wrongdoings in the present situation to another person, i.e., the communicative addressee. Interactants are communicatively skilled at portraying themselves, for instance, as calm, reasonable, and judicious, while the person whose indignation or reproaches are imitated is presented as arrogant, presumptuous or near hysterical (cf. Bergmann and Luckmann, 1999 for the empirical analysis).

The absence of a universal moral code on which interactants can depend is also visible in another phenomenon: Moral communication in our society is generally guided by the principle of *indirectness*. Respect and withdrawal thereof tend not to take place directly in our society, but rather indirectly, in subtle hints or mediated through others.

“Whether moralization takes place overtly or covertly seems to be essentially determined by the risk calculation of the actor. It is generally the case for moral communication that it is frequently characterized by a high degree of *indirectness*, i.e. only hints at the moral verdict and ‘sugarcoats’ it or passes it on by a detour through others.” (Bergmann and Luckmann, 1999, 31, our translation)

Interactants create this caution and indirectness in a variety of ways, for instance by asking seemingly innocuous questions (“The- the Theissens moved out, huh?”; cf. Bergmann, 1993, 85 for this example). A very effective means for creating indirectness are subtle hints and euphemisms. In an analysis of institutional communication (interviews for admission to psychiatric care), Bergmann showed how criticism can be softened: “Doctor Hollman told me something like you were running across the street not so completely dressed or something like that.” In this utterance from a psychiatrist addressing a patient, the directness of the remark is mitigated by several means and indirectness is created. Not only are there mitigators (“something like,” used twice); the utterance is also ascribed to another, absent, person (“Doctor Hollmann”). The one responsible for the claim is thus not the psychiatrist as a speaker, but another doctor. Most importantly, though, the litotes “not so completely dressed” serves to mitigate the face-threatening situation. Such use of litotes *avoids* drastic expressions by negating the opposite (the potentially face-threatening adjective ‘naked’ becomes ‘not so completely dressed’) (for analyses of this and other examples, cf. Bergmann, 1992, 143 ff.).

The avoidance of face-threatening actions is a central argument for Goffman’s analyses of face-to-face interactions. In moral communication there is an inherent threat to the face in Goffman (1967) sense. It is thus delicate ground to tread on for both parties, requiring them to use moral communication with caution. For this, indirectness can be created with a variety of linguistic means. Günthner shows that moral communication is often accompanied by expressions of affect. The affective charge can be visible in choice of words, but an important role is also played by *prosody*. Günthner (1996) discusses reproaches as an example. In the data, there is a remarkable number of reproaches taking the form of questions, often introduced with ‘why’. Günthner showed how the seemingly innocuous question, “Why did you say Konstanz?” through its prosodic realization becomes a reproach: “WARUM = *SA:↑↓GEN=SIE = DANN=KONSTANZ.” This why-question does not simply request a reason for the behavior; it represents the behavior displayed as inappropriate (the addressee of the reproach had mixed up two places). As Günthner (1996) shows through this and other examples, it is especially the prosody which turns this utterance into a reproach (thus also leading to an apology from the addressee). Günthner identifies these features (among others) which turn a question into a reproach: “global increase of loudness, high global pitch, a rise-fall on the accentuated syllable and verum focus” (Günthner, 1996, 281). Modal particles and specific lexical elements (not used in the above example) can play a role in revealing the reproach. The recognizability of the reproach *as* a reproach, in this and other examples, is carried almost exclusively by the affective charge of the prosody – the ‘bare’ question would not make the utterance a reproach; in fact, the ‘bare’ question sounds as though the speaker was making an innocent inquiry about the reason. However, the realization of the reproach in the form of a why-question also allows the addressee of the reproach to ignore the affective charge and simply treat the reproach as a ‘question’ to which a factual ‘answer’ can be given (“Because …”). For the originator of the reproach, the question format provides a way to retreat to the question character if necessary. In the case of counter-reproach, they can insist on just having asked a question (“I was just asking”).

It thus turns out that moral communication is certainly a dangerous business for interactants. This is because when speaking negatively about others, they inevitably put their own moral integrity at risk in that they may appear presumptuous, crass, condescending, etc. They also risk becoming themselves an addressee of moral communication, for instance by provoking a counter-reproach from the other person. The strategy of indirectness solves the problem of the inherent risk for interactants in moral communication; they also protect the actor from ‘counter-moralizing’.

### 3.3 Talking about the future: projective genres

Projective genres are understood as consolidated forms of communication targeted at the *future* – in other words, forms of speaking about what is to come (Ayaß, 2021, 2024). This can be the near future (in a moment, very soon, this evening), in the foreseeable future (tomorrow, the day after tomorrow, this summer) or in the distant future (some way down the road, in 10 years, one day). Projective genres include a wide range of communicative forms. What unites projective genres as a family is that they provide a solution to the common communicative problem of *engaging with the future*. In a broader sense, projective genres as a family of genres thus include all forms in which people plan the future and prepare action to execute this future – this may be a dinner being prepared, a vacation that should be planned and booked, or the building of a home that must be financed and projected. Projective genres are thus genres which envision a future. They refer to the anticipation, the planning, and creation of events to come.^4^ The nearer the goal of the plan comes, the more binding arrangements and promises become. Both minor and major projects will require some form of commitment. Especially a project that is complex and has an obligatory aim (e.g., a family celebration) makes it necessary to turn uncertainty into certainty and what is vague must become concrete in the course of the planning. At the very beginning, a project may still be a vague idea, but it must become incrementally more concrete if it is to be executed. At specific points in the planning process uncertainty must be turned into certainty and what is vague must become concrete.

For projective genres, the typical communicative phenomena are different from those, say, of genres of reconstruction or moral communication. For the projective genres, there appears to be an inventory in the internal structure which is specifically designed to engage with the future. A recurring element of the internal structure of projective genres are, for instance, *if-then* constructions. *If-then* constructions are particularly suitable because they allow distinctions between individual action steps (if A then B; but this also means first A then B), because they can be used to divide the future into phases (there is an A, and then there is a B) and because commitments are made (we will not be able to do B unless we have taken care of A). The grammatical structure chains up two or more actions in close proximity. Most importantly, however, *if-then* constructions create a temporality which places different states into a temporal sequence and links them consecutively. In projective genres, *if-then* constructions serve to represent the future. They anticipate and predict what will happen ‘then’. They structure the sequence of events in the future. Future goings-on and developments are broken down into individual steps and brought into a linear sequence – in the sense that here, too, one will not take the second before the first step. For actors, *if-then* constructions are a verbal means with which to reduce uncertainty in that they allow the anticipation and structuring of as yet unknown events. Others can then use them for orientation, contradict them, modify them, or simply confirm them. Schutz and Luckmann, in their analysis of projects of action, speak of a “more or less richly branched ‘decision tree’” (Schutz and Luckmann, 1989, 51) which a project of action can become. If progress is to be made at the ‘branches’ of a project of action, decisions must be made. *If-then* statements anticipate exactly this situation. They serve to reduce uncertainty and create commitment.

Another recurring element of the internal structure of projective genres and appears to be constitutive are *modal verbs*. Projects of action are connected to intentions, but at the same time subject to uncertainties, and they frequently encounter obstacles and constraints. By means of the modal verbs ‘want’, ‘should’, ‘can’, and ‘must’, interactants can articulate the room for action and its limitations. In projective genres, modal verbs take a central position within the interactive process. This is because *must’s* and *want-to’s*, as well as other modal verbs, contain structures of participation, assignments of responsibility, and expectations. They can impose constraints on the addressees or open up room for action. Modal verbs allow interactants to probe their own room for action, obtain permissions and concessions from one another and predict obstacles. For the procedure and the planning of the project of action, such interactive maneuvers are central. There are expectations as to what we can, should, must, and want to do which are distributed differently among the actors and which are articulated in the communication of concrete situations. They can be expressed as demands that somehow have to be met (“You must”); however, there are also options which contain room for action and choice-making (“Do you want to come or do you want to stay here”). In the interactive process of projective genres, modal verbs are also relevant for the progress of the project of action. This is because one person’s ‘want-to’ can become another person’s ‘must’. Modal verbs provide interactants with a possibility for continual mutual coordination so that the shared plan of action remains a shared one. Moreover, they periodically secure the cooperation. With the help of modal verbs, projective genres can mark different phases of projects of action. There are phases during which a project of action, or parts thereof, are in a *want-to* state (or a wish, an intention, a distant future) and other phases during which specific steps *must* be taken if the plan is to succeed. The more complex a project of action, the more progressive phases it will include, and the more modal verbs ‘help’ interactants to agree with each other and coordinate the different conditions of *want-to’s*, *must’s*, and *should’s* in which actors find themselves.

### 3.4 Ongoing and future developments of empirical genre analysis

The three mentioned research projects and the families of genres studied within them show two structural changes in their developments, pointing (a) to methodical/methodological developments and (b) to societal transformations. (a) The methodical/methodological developments that CA has undergone are of relevance also for genre analysis because it is methodologically linked to CA. In more recent projects, there is thus a shift in the data corpus from auditive recordings (used for example at the beginning of genre analysis in the project on reconstructive genres) to audiovisual recordings (used in the project on projective genres). As a consequence, different interactive phenomena come into focus and can be studied at a higher degree of complexity in the video material. Thanks to video recordings, for instance, the project on projective genres also looked at the ways interactants handled calendars, i.e., artifacts which play a major role for planning. Another project with a genre-analytical research question was concerned with representations and renditions of audiovisual presentations such as “Powerpoint” (Knoblauch, 2013) as *performative genres*. It analyzed spatial, physical and visual elements, such as the body of the presenter in the space between the audience and the slides. Thanks to video data, this project primarily demonstrated the role of pointing gestures as a constitutive feature in the internal structure. Video data, in principle, also allow a shift to videographical methods (see Knoblauch and Schnettler, 2012). (b) Connected to this are the transformations which the analyzed fields and their interactions experience chiefly because of the ubiquity of media. Their use is deeply embedded in everyday interactions. The project on projective genres, for example, revealed the enormous role played by social media not only for general everyday interaction, but *primarily* for the communication of plans and for communicating intentions. Such mediated interaction (e.g., writing text messages) can be analyzed empirically within genre analysis. Genre analysis of face-to-face communication especially benefits from analyses that are not restricted to the interaction through the medium, but shed light on their *embeddedness in everyday communication* (see Hitzler and Kramer, 2023 for an example). Genre analyses in the future will almost by necessity make references to the use of media and technical artifacts – simply because of their pervasiveness in everyday communication. The impact of this “everydayification” of media (see Ayaß, 2012) – their ubiquity, their routine use in everyday interaction – on genre analysis is that media become interwoven with everyday interaction. Although genre analysis is primarily concerned with face-to-face communication, the concept can also be applied to mediated communication, such as in social media, especially in situations where actors interact, e.g., by exchanging text messages. However, it should remain clear that the application of genre analysis to written forms of communication has its limits: The philological analysis of genre as known from literary or film studies is not an analysis of communicative genres in the sociological sense. The close connection between genre analysis and CA is no coincidence. The aim of the analysis of communicative genres is to demonstrate the situational realization of these genres and the interactive orientation of those involved in these established solutions to communicative problems. So, for genre analysis, an orientation and alignment with CA is therefore essential.

## 4 Discussion: Conversation Analysis, genre analysis and sociology

From the above elaborations, it probably has become clear that the aims of genre analysis go beyond questions of CA. Genre analysis is not merely an analysis of ‘large’ sequences, but links the empirical analysis of consolidated communicative forms – i.e. the communicative genres – with the question of communicative problems solved by the genres. Genre analysis thus has the potential to make CA attractive for sociology beyond ethnomethodology in the narrower sense. This is relevant in several respects.

(1) The first one is the level of sociological sub-disciplines: It has already been demonstrated that genre analysis is situated in a framework of the sociology of knowledge. Communicative genres allow the transmission of knowledge (about the past, about the future, about what is to be seen as right and wrong, etc.), and they are themselves anchored in the stocks of knowledge of the interactants. Furthermore, the *sociology of language* receives crucial impulses from genre analysis. Thomas Luckmann, already in the 1970s, had turned to the sociology (and the philosophy) of language (e.g., Luckmann, 1973, 1979). These texts testify to Luckmann’s great interest in such thinkers as Wilhelm von Humboldt (especially the “Kawi Essay,” a work on the Kawi language of the island of Java, published posthumously in 1836), Roman Jacobson, Mikhail M. Bakhtin and Valentin N. Vološinov, and their writings on the connections between language and society. Luckmann was irritated by the fact that linguistics and sociology were all but unaware of each other. In retrospect, he described this relationship as thus: “in fact, it seemed that they existed in separate universes.” Especially sociology and the sociology of language were “linguistically naïve to the point of ignorance” (Luckmann, 2013, 42). Genre analysis closes this gap, completing and strengthening the sociology of language. Genre analysis understands itself as a contribution to the connection between language (and interaction) and society. It creates this link explicitly through the nexus of the internal and external structures. Moreover, the analyses of the different families of genres allow connections to other research areas established in sociology. In genres, the social presence is negotiated, the past and the future are discussed. For example, the analysis of reconstructive genres makes a contribution to the analysis of the communicative fabrication, memory and remembrance in everyday communication (Halbwachs); the analysis of the genres of moral communication was able to show how the moral composition of a society is communicated (Durkheim, Goffman); finally, the analysis of projective genres merges with action theory (Schutz, Luckmann) and the sociology of time (Merton, Sorokin). To these and other research areas, genre analysis contributes empirical insights showing how these social phenomena (i.e., memory, time, etc.) are created communicatively and made relevant in everyday situations. Finally, the fact that a specific communicative form is solidified into a genre is an indication of the structure of relevance in society (Schutz, 2011).

(2) Secondly, genre analysis can provide a gateway for CA to sociological theories, especially to the “Social Construction of Reality” (Berger and Luckmann, 1966) and the associated approaches. From a sociological perspective, CA is an ethnomethodological undertaking (and will always be). From the outset, however, genre analysis essentially connects (conversation) analysis with a theoretical question. This is coherent in every respect given the origin of genre analysis. A look at the theoretical profiles of the two originators of the analysis of communicative genres shows this very clearly. For Bergmann, the determining element in CA, from the beginning, is the ethnomethodological question about the “ongoing accomplishment” of social reality. In Luckmann, too, there is a conceivable path from his (and Peter Berger’s) theoretical considerations in the “Social Construction of Reality” (Berger and Luckmann, 1966) to an interest in the empirical study of interactions from the 1980s. For, even if not all human activity is communicative in a narrow sense, activities are all somehow accompanied by communication. “Human social reality and the world view that motivates and guides interaction is mainly constructed in communicative processes” (Luckmann, 2013, 44). Knoblauch (2020), building on Berger/Luckmann, explicitly speaks of genre analysis as “communicative construction of reality,” emphasizing the sequentiality of human action. CA and the typical records it creates now provide the means to analyze meticulously these processes of creation as they unfold situatively. Luckmann shows his interest in the analysis of genres as thus: “I wish to see how social reality is constructed, reconstructed, and how this happens in detail. This is social construction *en detail. En detail*!” (Luckmann, 2012, 30; our translation). The sequential procedure in CA allows for a step-by-step analysis of the interactants’ actions, thus exposing, turn by turn, the layers of meaning in its production. CA is the methodical tool *par excellence* to reveal these processes of constructing social reality analytically. And yet, to be precise, CA is (just) the means, not the end in this process. This is because genre analysis is not concerned solely with the interactive structures and the orderliness of social interaction; it is concerned with the communicative procedures in which these interactive structures and this orderliness generate and communicatively mediate reality. Seen in this way, the analysis of communicative genres is an empirical answer to the question of how social reality is constructed.

(3) Finally, genre analysis allows the connection with concepts in social theory as relevant for numerous sociological approaches such as ethnomethodology (Garfinkel) or the “Social Construction of Reality” (Berger and Luckmann, 1966), which draw on Alfred Schutz and his writings on the essential intersubjectivity of the social world. In carrying out communicative genres, interactants rely on shared stocks of knowledge. As demonstrated above, communicative genres are consolidated forms of creating and mediating social reality. For this, genres provide consolidated solutions to problems, which in their consolidation become *manifest*. These processes are observable in their practical execution to actors as completed, objectified reality. Communicative genres stabilize communicative situations in that they create communicative sequences that are predictable for and *jointly* created by interactants reciprocally. Communicative genres are a form of intersubjectively constituting reality whereby interactants reciprocally clarify the character of the current situation. Genres are thus also means for the *creation* of intersubjectivity, which plays a crucial role for interactions in general (Lindström et al., 2021). In the joint realization of genres, interactants signal to each other that they are members of the same social reality which they share and generate together. Genres are determined by social structures which provide the external conditions for language and interaction to articulate themselves in the first place (i.e., the external structure). Conversely, communicative acts and genres (and their internal structure) impact social structures and have the potential to change them. “Languages, social structures and communicative acts continue to ‘determine’ one another, resulting in new ‘syntheses’ in the real lives of real people” (Luckmann, 1992, 222).

## Author contributions

The author confirms being the sole contributor of this work and has approved it for publication.

## Footnotes

^1^Parts of this paper are based on (Ayaß 2011, 2021, 2024). I am, again, deeply indebted to Ruben Bieker, Wetzlar, Germany, for his accurate translation.

^2^This is neither a claim that these are the only families of genres, nor that they are the only central ones. Luckmann also counts genres of upbringing (Luckmann, 2012, 35) among those that are probably of relevance for all societies.

^3^The specific research projects are: (1) DFG project “Strukturen und Funktionen von rekonstruktiven Gattungen der alltäglichen Kommunikation” (Structures and functions of reconstructive genres of everyday communication). Supervisors: Jörg Bergmann, Thomas Luckmann, University of Konstanz, 1984–1989. Research staff members: Angela Keppler, Hubert Knoblauch, and Bernd Ulmer. (2) DFG project “Formen der kommunikativen Konstruktion von Moral. Gattungsfamilien der moralischen Kommunikation in informellen, institutionellen und massenmedialen Kontexten” (Forms of the communicative construction of morality: families of genre of moral communication in informal, institutional and mass-media contexts). Supervisor: Jörg Bergmann, Thomas Luckmann, Universities of Gießen and Konstanz, 1992–1997. Research staff: Ruth Ayaß, Verena Blöcher, Gabriela B. Christmann, Michaela Goll, Susanne Günthner, and Kirsten Nazarkiewicz. (3) DFG project “Planning-in-Action: Die kommunikative Verfertigung von Zukunft in projektiven Gattungen” (Planning in Action: the communicative construction of the future in projective genres). Supervisor: Ruth Ayaß, University of Bielefeld, 2021–2024. Research staff: Sarah Hitzler, Jonas Kramer, and Ajit Singh.

^4^In the context of projective genres, this refers to something else than the planning or the (mental) projection of *communication*, as described by Luckmann (1995) as “interaction planning” or by Linell (1998) as “communicative projects.”
